# Psychological predictors of CPAP therapy adherence in obstructive sleep apnea patients: insights from the predisposing, precipitating, and perpetuating factors model

**DOI:** 10.1016/j.ijchp.2025.100602

**Published:** 2025-06-24

**Authors:** Valentina Poletti, Elvia Battaglia, Eleonora Volpato

**Affiliations:** a IRCCS Fondazione Don Carlo Gnocchi, Via Capecelatro, 66, 20148, Milan, Italy; b Department of Psychology, Università Cattolica del Sacro Cuore, Largo Gemelli, 1, 20123, Milan, Italy; c Sleep Centre, IRCCS Fondazione Don Carlo Gnocchi, Via Capecelatro, 66, 20148, Milan, Italy; d Sleep Centre, Centro Diagnostico Italiano (CDI), Via Saint Bon, 20, 20147, Milan, Italy; e Research Group Health Psychology, University of Leuven, Leuven, Belgium

**Keywords:** Obstructive sleep apnea (OSA), CPAP adherence, Psychological predictors, 3P model, Patient-centered interventions, Rehabilitation psychology

## Abstract

Continuous positive airway pressure is the standard treatment for obstructive sleep apnoea (OSA), a condition marked by recurrent interruptions in breathing during sleep that impairs quality of life. Despite its efficacy, adherence to continuous positive air pressure (CPAP) remains suboptimal and is influenced by various psychological and contextual factors.

This scoping review adopts the 3P model—predisposing, precipitating, and perpetuating factors—to identify key motivators and barriers impacting CPAP adherence in OSA patients. A systematic search of PubMed, PsycINFO, and Scopus identified 43 relevant studies. Inclusion criteria focused on peer-reviewed, full-text articles investigating psychological aspects influencing CPAP adherence in adults with OSA. Paediatric populations, non-English publications, and studies without an explicit focus on psychological variables were excluded.

Predisposing factors include psychological comorbidities, low health literacy, and misconceptions about OSA and CPAP. Perpetuating factors include ongoing psychological barriers, inadequate patient education, and suboptimal communication with healthcare providers. Precipitating factors include device-related anxiety, and perceived stigma.

In addition, motivators that support adherence have been identified, such as perceived improvements in quality of life, bed partners’ support, and tailored educational programmes highlighting the benefits of CPAP. Interventions such as cognitive behavioural therapy and psychological patient support show promise in improving adherence. Introducing a novel application of the 3P model, this scoping review underscores the complexity of psychological and behavioral determinants of CPAP adherence, highlighting the need of a multifaceted, patient-centered approach. Future research should evaluate the effectiveness of personalized interventions through longitudinal studies to assess their impact on treatment adherence and clinical outcomes.

## Introduction

Obstructive sleep apnoea syndrome (OSA) is the most common sleep-related breathing disorder (SBD), with a prevalence, defined by an apnoea-hypopnoea index (AHI) ≥5 (Berry et al., 2017), estimated to average 22% (range: 9-37%) in men and 17% (range: 4-50%) in women (Franklin & Lindberg, 2015). OSA involves repeated episodes of airway obstruction during sleep, causing apnoeas and hypopnoeas (Young et al., 2012), that severely impact quality of life (W. Lee et al., 2016). People with OSA often report excessive daytime sleepiness, occasionally accompanied by sudden sleep episodes (Lal et al., 2021), and cognitive impairments, particularly in episodic memory and information processing speed (Beaudin et al., 2021). These issues can severely compromise social relationships, workplace performance, and psychological well-being (Scarpina et al., 2021). Despite this, some patients, known as 'mis-perceivers', may not fully recognize their symptoms (Lee et al., 2022). Reduced symptom awareness, limited health literacy among patients (Ellender et al., 2021) and inadequate knowledge of the condition among primary care physicians (Devaraj, 2020), contribute significantly to the high rates of underdiagnosis and undertreatment, further amplifying the associated health risks (Alterki et al., 2023). To date, continuous positive airway pressure (CPAP), a device that delivers pressurized air to keep the airways open during sleep, is considered the gold standard treatment for OSA (Sánchez-De-La-Torre et al., 2023). The initial period of CPAP use, often referred to as the adaptation phase, typically encompasses the first few weeks of treatment, during which patients undergo mask fitting, pressure titration, and receive educational and behavioral support to facilitate adjustment and promote adherence (Ward et al., 2014). However, adherence to therapy, defined by guidelines as using the device for at least 4 hours per night (Weaver, 2019), remains low, with nearly 50% of patients failing to meet this threshold (Sawyer et al., 2011). The most commonly identified causes of poor adherence to CPAP therapy include physical discomfort (Tiyapun et al., 2023) and low income (Palm et al., 2021), which has been associated with significantly shorter nightly CPAP use, likely due to financial strain, limited health literacy, reduced access to supportive follow-up services, and in some countries, the complete absence of a national healthcare system (NHS). In addition to that, a growing body of research in health psychology emphasizes that treatment adherence is also strongly influenced by subjective factors, such as patient attitudes, subjective norms, self-efficacy, and perceived behavioral control, which can act as either risk factors or protective factors for treatment adherence depending on the circumstances (Horne & Weinman, 2020).

### Conceptual framework and scope of the current contribution

Understanding the factors that contribute to the development and persistence of health-related behaviors is critical for improving diagnosis, treatment adherence, and long-term outcomes (Offord & Kraemer, 2000). The 3P Model—originally proposed by Spielman and collaborators (Spielman et al., 1987) to explain the onset and maintenance of insomnia—offers a comprehensive framework for conceptualizing these factors by categorizing them as predisposing, precipitating, and perpetuating. To date, the model has since been applied to a range of psychological and behavioral conditions, offering valuable insights into the interplay of individual, contextual, and systemic influences on health behaviors (Wright et al., 2019).

The 3P Model identifies predisposing factors as underlying vulnerabilities that increase susceptibility to a given condition or behavior. These factors may include genetic predispositions, personality traits, or long-standing beliefs and attitudes. Precipitating factors are acute events or changes that trigger the onset of a condition or behavior, such as life stressors, environmental disruptions, or significant transitions. Finally, perpetuating factors are those that sustain a condition or behavior over time, often by interfering with resolution or recovery. These may include maladaptive coping strategies, insufficient support systems, or barriers within the healthcare environment (Durayappah, 2011).

This scoping review applies the 3P framework to the study of CPAP adherence in patients with OSA. By categorizing the psychological and behavioral determinants of adherence through the lens of the 3P Model, the review aims to deepen the understanding of adherence behaviors and identify opportunities for intervention.

## Methods

Following PRISMA-ScR checklist (Page, O. N., 2024), a scoping review was conducted to provide a critical analysis of the literature on psychological factors influencing CPAP adherence in patients with OSA. A scoping review is a type of literature review aimed at mapping the existing body of research on a particular topic or field, highlighting key concepts, theoretical frameworks, sources, and research gaps (Munn et al., 2018). Unlike systematic reviews, it typically does not evaluate the quality of the studies included or provide detailed syntheses. Instead, it offers a comprehensive overview of the scope and diversity of evidence, serving as a foundation for guiding future research, policy development, and practice (Arksey & O’Malley, 2005).

### Identifying the research question

The Population, Concept, and Context (PCC) framework (Peters et al., 2015) was followed to shape the research question and inclusion criteria. The primary research question guiding this scoping review was: *What are the psychological and behavioral factors influencing adherence to CPAP therapy in patients with OSA, as conceptualized within the 3P Model framework?*

### Search strategy and eligibility criteria

Systematic literature searches were performed from August to December 2024, covering studies published from the inception of the selected databases up to January 2025. The PubMed, Scopus, and APA PsycInfo databases were searched to identify all relevant studies. Outcomes for each database were imported into Zotero (Ahmed & Al Dhubaib, 2011), a free web-based collaborative software platform designed for organizing selected articles.

Peer-reviewed articles written in English were included if they examined the factors, with a specific focus on psychological variables, influencing CPAP adherence in adult populations diagnosed with OSA, using either quantitative or qualitative methodologies. Exclusion criteria encompassed articles not written in English, single case reports, studies conducted on pediatric populations, and those involving conditions other than OSA. The following Boolean search strategy was employed to query titles and abstracts: CPAP *OR* continuous positive airway pressure *AND* treatment adherence *OR* CPAP adherence *AND* barriers *OR* motivators.

### Selection and data collection process

The initial search and independent screening of all titles and abstracts were conducted by VP. The selected articles were cataloged in a Microsoft Excel database and subsequently shared with EV, who performed a second review to ensure accuracy. Any discrepancies were resolved with the involvement of EB. During the initial title screening, only records that were clearly unrelated to the topic—such as studies focusing on other clinical conditions (e.g., acute lung injury), studies conducted on animal models, or interventions unrelated to CPAP adherence—were excluded. This step was necessary due to the inclusion of broad search terms (e.g., "barriers", "motivators") that retrieved many non-specific records. Following this, VP, under EV's supervision, systematically categorized the factors identified in each selected article, assigning them to the three categories of the 3P Model (predisposing, precipitating, and perpetuating factors). Finally, the results were synthesized and discussed by VP and EV, under the supervision of EB.

### Data extraction

For each article, the following information were extracted: author and year of publication, country, methodology (quantitative/qualitative), number of participants, diagnosis severity, specifying whether they were CPAP-adherent or CPAP-naïve (patients who have never used CPAP treatment), percentage of male participants, mean age and mean BMI of the sample, CPAP air pressure settings, adherence time, psychological barriers and motivators identified in the study. Subsequently, the identified factors were categorized into the three classes of the 3P Model and divided into barriers and motivators, defined as factors that respectively hinder or facilitate adherence to CPAP therapy.

## Results

**Fig. 1:**
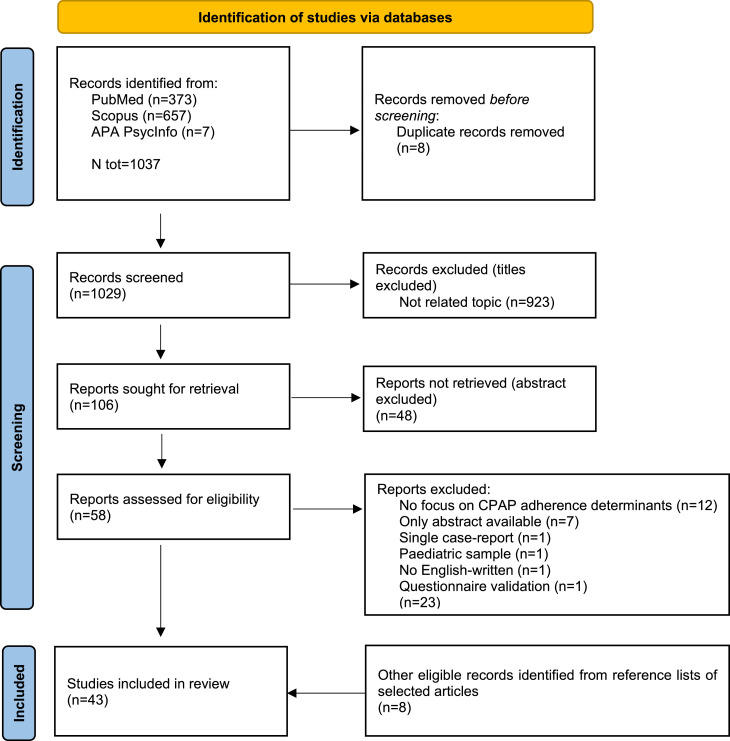
PRISMA 2020 flow diagram for studies’ selection process.

The study selection process is illustrated in Fig. 1. An initial search of the databases yielded 1037 potentially relevant studies. Following the identification and removal of 8 duplicate records, 1029 studies were considered for further screening. After evaluating titles and abstracts, and excluding materials that were not relevant, 58 articles were selected for a full-text review. A detailed examination of these articles led to the exclusion of 23 studies due to various specified reasons. Consequently, a total of 43 studies met the inclusion criteria, including 8 identified through reference list searches of the selected articles.

### Study descriptions

A total of 43 studies investigating the barriers and facilitators to CPAP use among patients with OSA were included. Most of the studies were conducted in the United States (18 studies, representing 41.86% of the total). Twenty-five studies (58.14%) utilized quantitative methodologies, while 18 studies (41.86%) employed qualitative approaches, such as semi-structured interviews and focus groups. The included studies involved 28,194 patients with OSA, of whom 2,289 were CPAP-naive (8.12%) and 25,905 were CPAP adherent (91.88%), as well as 1,592 bed partners and 24 primary care physicians. Table 1 reports more detailed information about the included studies.

**Table 1:** Included studies characteristics.

Author	Country	Methodology	Intervention	Subjects	Males (N, %)	Diagnosis	OSA screening tool	OSA Severity	Age (m±sd)	BMI (m±sd)	Auto-CPAP	cmH2O (m±sd)	CPAP adherence	Timepoint of Adherence Assessment
Bakker et al. 2011 (Bakker, O’Keeffe, Neill, et al., 2011)	New Zealand	Quantitative	Standardized 4-week CPAP trial with education, follow-up calls, and scheduled visits	126 CPAP-naïve patients	100 (79.36%)	OSA	ESS	Severe	51.01 ± 11.64	38.35 ± 9.12	Auto-CPAP (7 days titration), then fixed-pressure CPAP	11.1 ± 3.1	5.63 hours/night (interquartile range [IQR] 2.55)	4 weeks (follow-up at 2 and 4 weeks)
Bakker et al. 2014 (Bakker, O’Keeffe, …, et al., 2014)	New Zealand	Qualitative [Focus Group]	Standardized CPAP initiation with education, follow-up call, and 2- & 4-week reviews	18 CPAP patients	11 (61.11%)	OSA	ESS	/	47 ± 10.25	/	Not specified (likely fixed-pressure CPAP)	13.8 ± 4.2	6.32 ±1.25 hours/night	4 weeks (end of supervised trial)
Berg et al. 2023 (Berg et al., 2023)	USA	Qualitative [Semi-structured Interviews]	No	15 CPAP patients and their bed partners	3 (20%)	OSA	ESS	Severe	47.4 ± 8.9	44.8 ± 11.4	Not specified (likely fixed-pressure CPAP)	9.4 ± 3	5.5 ± 1.6 hours/night	Not specified but mean 2.6 years on CPAP
Billings et al. 2011 (Billings et al., 2011)	USA	Quantitative	Randomized trial comparing home vs. lab-based CPAP initiation with standardized protocols	191 CPAP-naïve patients	124 (65%)	OSA	ESS	Severe	48 ± 12	38.93 ± 1.32	Not specified (likely fixed-pressure CPAP)	10.8 ± 0.29	Average nightly use in min (1 month): Black Non-Hispanic151 ± 103 White Non-Hispanic: 256 ± 130 Average nightly use in min (3 months): Black Non-Hispanic: 179 ± 106 White Non-Hispanic: 267 ± 141	1 and 3 months
Broström et al. 2007 (Broström et al., 2007)	Sweden	Quantitative	No	247 CPAP patients	203 (82.19%)	OSA	Not specified	Severe	60.1 ± 9.2	33.2 ± 10	Not specified (likely fixed-pressure CPAP)	9.5 ± 2.4	Type D: 292.1±138.4 minutes Non-Type D: 378.2±116.6 minutes	Retrospective assessment (mean 55 months on CPAP)
Broström et al. 2010 (Broström et al., 2010)	Sweden	Qualitative [Semi-structured Interviews]	No	23 CPAP patients	13 (56.52%)	OSA	Not specified	Severe	60.5 ± 17	35.5 ± 11		9.6 ± 2.1	Men: 302.2 ± 132.4 minutes Women: 298.2 ± 138.4 minutes	
Broström et al. 2021(Broström et al., 2021)	Sweden	Qualitative [Semi-structured Interviews]	No	24 Primary Care Physicians	6 (25%)	/	Not specified	/	/	/	Not specified (likely fixed-pressure CPAP)	/	/	Not specified
Buckingham et al. 2020 (Buckingham et al., 2020)	Australia	Quantitative	No	110 CPAP-naïve patients	67 (60.91%)	OSA	Not specified	/	58.28 ± 14.61	/		/	345.44 ± 135.39 minutes	
Bulteel et al. 2020 (Bulteel et al., 2020)	France	Qualitative [Semi-structured Interviews]	No	17 CPAP patients	17 (100%)	OSA with spinal cord injury	Not specified	/	62 ± 4.75	27 ± 3	Not specified (likely fixed-pressure CPAP)	/	5.5 ± 2.9 hours/night	Not specified
Choi et al. 2022 (Choi et al., 2022)	Korea	Quantitative	No	246 CPAP-naïve patients	208 (84.55%)	OSA	Not specified	Severe	51.30 ± 11.40	26.55 ± 3.5	Auto-PAP (better adherence) and fixed-pressure CPAP	/	6 months: 5.05±0.17 hours/night 9 months: 4.77±0.18 hours/night	6 and 9 months
Collen et al. 2012 (Collen et al., 2012)	USA	Quantitative	No	90 CPAP-naïve patients	90 (100%)	OSA	ESS	Moderate/Severe	39.9 ± 11.2	27.9 ± 8.0	Not specified (likely fixed-pressure CPAP)	/	Non-PTSD: 4.2 ± 2.1 PTSD: 2.5 ± 1.8	Not specified (retrospective assessment)
Corrigan et al. 2022 (Corrigan et al., 2022)	Canada	Quantitative	No	242 CPAP patients	/	OSA	ESS	/	51 ± 13	/		/	3.19 (2.8–3.58) hours/night	
Daabek et al. 2021 (Daabek et al., 2021)	France	Quantitative	No	294 CPAP patients	189 (64.3%)	Chronic respiratory failure in OSA	Not specified	/	83 ± 28.2	30.8 ± 10	Not specified (likely fixed-pressure CPAP)	/	7.3 [5.4; 8.8] hours/night	≥12 months (mean duration not specified)
Dzierzewski et al. 2016 (Dzierzewski et al., 2016)	USA	Quantitative	No	191 CPAP patients	191 (100%)	OSA	Not specified	/	58.86 ± 10.96	/		9.48 ± 2.70	/	
Edmonds et al. 2015 (Edmonds et al., 2015)	USA	Quantitative	No	68 CPAP patients	36 (53%)	OSA	Not specified	Severe	49 ± 11.4	38.6 ± 9.9	Not specified (likely fixed-pressure CPAP)	/	4.5 ± 2.7 hours/night	1 week and 1 month
El-Solh et al. 2011(El-Solh et al., 2011)	USA	Quantitative	No	296 CPAP patients	296 (100%)	OSA	ESS	Moderate/Severe	60.6 ± 8.1	35.15 ± 6.6	Not specified (likely fixed-pressure CPAP)	/	Adherent: 422 ± 76 minutes Non-Adherent: 226 ± 94 minutes	1 month
Fung et al. 2017 (C. H. Fung et al., 2017)	USA	Qualitative [Focus Group]	No	35 OSA patients [of these: 27 CPAP patients]	30 (86%)	OSA	Not specified	/	23 (66%): 65–69 years 7 (20%): 70–74 years 5 (14%): 75 years or older	/	Not specified (likely fixed-pressure CPAP)	/	/	Not specified
Fung et al. 2017 (C. Fung et al., 2017)	USA	Quantitative	No	564 CPAP patients	477 (85%)	OSA	Not specified	/	71.4 ± 5.9	/	Not specified (likely fixed-pressure CPAP)	/	5.2 ± 2 hours/night	<36 months
Gagnadoux et al. 2011 (Gagnadoux et al., 2011)	Chile	Quantitative	No	1141 CPAP patients	/	OSA	ESS	Severe	≥ 65	/	Not specified (likely fixed-pressure CPAP)	/	/	mean: 504 days (range: 91-1035)
Gentina et al. 2019 (Gentina et al., 2019)	France	Quantitative	No	290 CPAP patients	224 (77.24%)	OSA	Not specified	Severe	53±16	32.1±7.3	Not specified (likely fixed-pressure CPAP)	/	/	120 days
Goldstein et al. 2022 (Goldstein et al., 2022)	USA	Qualitative [Semi-structured Interviews]	No	30 CPAP patients	27 (90%)	OSA	Not specified	Moderate/Severe	58 ± 17	about 29	Not specified (likely fixed-pressure CPAP)	/	/	Not specified
Goyal et al. 2017(Goyal et al., 2017)	India	Quantitative	No	79 CPAP patients	58 (73.42%)	OSA	ESS; STOP Bang Questionnaire	Severe	54±17	about 35	Not specified (likely fixed-pressure CPAP)	/	/	Not specified
Graco et al. 2019 (Graco et al., 2019)	Australia	Qualitative [Semi-structured Interviews]	Initiation of auto-CPAP therapy with one-month support; adherence and experiences assessed over 12 months.	80 CPAP-naïve patients	13 (81.3%)	OSA in chronic tetraplegia	Not specified	Severe	56.3 ± 15.5	27.2 ± 5.7	Auto-CPAP	/	One month: 3.1 h (SD = 2.5; 38% adherent); 6 months: 2.6 h (SD = 2.8; 25% adherent); 12 months were 2.1 h (SD = 3.2; 25% adherent)	1, 6, 12 months
Guralnick et al. 2012 (Guralnick et al., 2012)	USA	Quantitative	Preoperative CPAP initiation with education and auto-titrating device	211 CPAP-naïve patients	130 (61.6%)	OSA	STOP-Bang Questionnaire	Moderate/Severe	61.9 ± 11.0	33.5 ± 8.1	Not specified (likely fixed-pressure CPAP)	8.8 ± 2.0	About 2.5 hours7night	Perioperative period
Han et al. 2022 (Han et al., 2022)	Australia	Quantitative	No	1188 CPAP patients	/	OSA	Not specified	Severe	range: 21–89 years old >75 years old: n=81 (7,8%)	about 30	Not specified (likely fixed-pressure CPAP)	/	<75 years old: 4.53 ± 2.42 > 75 years old: 4.38 ± 2.55	End of adaptation period
Khan et al. 2019 (Khan et al., 2019)	USA	Qualitative [Semi-structured Interviews]	Semistructured motivational interviews with patients and caregivers during educational sessions	28 CPAP patients	12 (43%)	OSA	Not specified	/	58 ± 11.75	35.5 ± 7.2	Not specified (likely fixed-pressure CPAP)	/	/	Not specified
Khot et al. 2022 (Khot et al., 2022)	USA	Qualitative [Qualitative Checklist]	Intensive CPAP adherence protocol during stroke rehabilitation, including early desensitization and self-efficacy training.	62 CPAP-naïve patients	32 (61.5%)	OSA in acute ischemic stroke or intracerebral hemorrhage	Not specified	/	59 ± 10.8	30.4 ± 6.8	Not specified (likely fixed-pressure CPAP)	/	4.7 ± 2.6 hours/night	3 month
Laharnar et al. 2024 (Laharnar et al., 2024)	Germany	Quantitative	No	1546 CPAP bed partners	1122 (72.6%) women	OSA	Not specified	/	59.9 ± 11.5	/	Not specified (likely fixed-pressure CPAP)	/	/	Not specified
Luyster et al. 2016 (Luyster et al., 2016)	USA	Qualitative [Focus Group]	Couples-oriented education and support intervention to enhance PAP adherence	14 CPAP patients and 11 bed partners	patients: 9 (64%); bed partners: 3 (27.2%)	OSA	Not specified	Moderate	Patients: 55.6 ± 10.3 Bed partners: 53.5 ±16.6	/	Not specified (likely fixed-pressure CPAP)	/	5–6 hours/night	Not specified
Milinovic et al. 2024 (Milinovic et al., 2024)	Croatia	Quantitative	No	87 CPAP-naïve patients	67 (77%)	OSA	ESS; SAQLI	Severe	55.6 ± 12.5	33.2 ± 7.4	Not specified (likely fixed-pressure CPAP)	/	5.1 ± 1.9 hours/night	1 month
Møkleby et al. 2019 (Møkleby & Mengshoel, 2019)	Norway	Qualitative [Semi-structured Interviews]	No	7 CPAP patients	5 (71.43%)	OSA	Not specified	/	/	/	Not specified (likely fixed-pressure CPAP)	/	/	Not specified
Nagarajan et al. 2012 (Nagarajan et al., 2012)	India	Qualitative [Semi-structured Interviews]	No	343 CPAP-naïve patients	/	OSA	Not specified	76.1% severe	/	/	Not specified (likely fixed-pressure CPAP)	/	/	6 months
Palm et al. 2021 (Palm et al., 2021)	Sweden	Quantitative	No	20,521 CPAP patients	14501 (70.7%)	OSA	ESS	Severe	57.8 ± 12.2	32.0 ± 6.1	Not specified (likely fixed-pressure CPAP)	/	355 minutes (interquartile range, 240-420 min)	Mean 1.3 ± 0.8 years
Rapelli et al. 2022 (Rapelli et al., 2022)	Italy	Qualitative [Focus Group]	No	32 CPAP patients	23 (63%)	OSA	Not specified	/	59.61 ± 11.18	≥30	Not specified (likely fixed-pressure CPAP)	/	/	Not specified (during 1-month inpatient rehabilitation)
Rezaie et al. 2018 (Rezaie et al., 2018)	Iran	Qualitative [Semi-structured Interviews]	No	97 CPAP patients	79 (81.44%)	OSA	Not specified	Severe	48.76 ± 12.4	30.96 ± 5.07	Not specified (likely fixed-pressure CPAP)	/	/	First 2 weeks
Sawyer et al. 2010 (Sawyer et al., 2010)	USA	Qualitative [Semi-structured Interviews]	No	15 CPAP patients	13 (87%)	OSA	Not specified	Severe	53.9 ±12.7	/	Not specified (likely fixed-pressure CPAP)	10.7 ± 1.6	4.98 ± 0.5 hours/night	First week of treatment
Simon-Tuval et al. 2009 (Simon-Tuval et al., 2009)	Israel	Quantitative	No	162 CPAP-naïve patients	120 (74.7%)	OSA	Not specified	Severe	54.9 ± 12.0	32.3 ± 5.4	Not specified (likely fixed-pressure CPAP)	/	/	Not specified
Tarasiuk et al. 2012 (Tarasiuk et al., 2012)	Israel	Quantitative	Financial subsidy for CPAP device to increase acceptance among low-SES patients	258 CPAP-naïve patients	/	OSA	ESS	Severe	50.8 ± 10.6	32.3 ± 6	Not specified (likely fixed-pressure CPAP)	/	/	12 months
Wallace et al. 2013 (Wallace et al., 2013)	USA	Quantitative	No	248 CPAP patients	120 (94%)	OSA	ISI	Severe	59 ± 11	33 ± 5	Not specified (likely fixed-pressure CPAP)	9.8 ± 2.8	3.2 ± 2.8 hours/night	Not specified
Wang et al. 2012 (Y. Wang et al., 2012)	China	Qualitative [Semi-structured Interviews]	Randomized controlled trial; combined patient education and progressive muscle relaxation	193 CPAP patients	162 (83.94%)	OSA	ESS, PSQI	Severe	51.91 ± 10.10	/	Not specified (likely fixed-pressure CPAP)	12.0 ± 3.58	/	4, 8, and 12 weeks
Wickwire et al. 2010 (Wickwire et al., 2010)	USA	Quantitative	No	232 CPAP-naïve patients	129 (56.5%)	OSA	Not specified	Severe	53.6 ± 12.4	34.4 ± 7.7	Not specified (likely fixed-pressure CPAP)	/	258.1±144.1 minutes	Not specified
Ye et al. 2012 (Ye et al., 2012)	USA	Quantitative	No	91 CPAP-naïve patients	49 (53.8%)	OSA	ESS, FOSQ	Severe	49.0 ± 12.0	39.2 ± 10.5	Auto-CPAP	/	Black people: 2.7 ± 2.2 hours/night Non-black people: 4.4 ± 2.9 hours/night	First week of treatment
Ye et al. 2017 (Ye et al., 2017)	USA	Qualitative [Interviews]	No	20 CPAP patients and their bed partner	11 (55%)	OSA	Not specified	Moderate	Patients: 49.6 ± 9.6 Bed partner: 50.1 ± 10.1	/	Not specified (likely fixed-pressure CPAP)	/	4.8 ± 2.0 hours/night	Not specified

### The 3P model factors in CPAP adherence

The analysis identified key motivators and barriers within the 3P Model influencing adherence to CPAP therapy (for a detailed overview, see Table 2). Among predisposing factors, knowledge about OSA management and CPAP emerged as the most frequently reported motivator (n=9) while a lack of such knowledge was the most cited barrier (n=10). Precipitating factors emphasized the feeling of anxiety induced by CPAP (n=10) and the social embarrassment related to device use (n=6). Perpetuating factors included perception of CPAP efficacy in symptoms resolution (n=13) and feeling supported from technical provider/medical staff (n=6) as motivators, whereas barriers such as lack of support from technical provider/medical staff (n=5) and impediment to intimacy (n=5) were highlighted. For a comprehensive overview of additional factors, refer to Table 2.

**Table 2:** Predisposing, perpetuating and precipitating factors in selected articles.

3 P Model	Motivators and Barriers	Factors	Number of Studies	Studies
Predisposing	Motivators	Knowledge about OSA management and CPAP	9	Bakker et al. 2011; Broström et al. 2021; Bulteel et al. 2020; Fung et al. 2017; Graco et al. 2019; Khan et al. 2019; Milinovic et al. 2024; Rapelli et al. 2022; Sawyer et al. 2010
		Trust in medical staff	5	Broström et al. 2010; Buckingham et al. 2020; Khan et al. 2019; Møkleby et al. 2019; Sawyer et al. 2010
		Self-Efficacy	4	Dzierzewski et al. 2016; Khot et al. 2022; Wallace et al. 2013; Ye et al. 2012
		Feeling supported from family and friends	3	Bakker et al. 2014; Broström et al. 2010; Gentina et al. 2019
		Sense of guilt for the bed partner	3	Broström et al. 2010; Bulteel et al. 2020; Møkleby et al. 2019
		Adopting a patient-centered approach	3	Broström et al. 2021; Milinovic et al. 2024; Rapelli et al. 2022
		Having a positive model	3	Khan et al. 2019; Rapelli et al. 2022; Simon-Tuval et al. 2009
		High motivation	1	Luyster et al. 2016
		Active health management engagement	1	Bakker et al. 2014
		Health consequences of OSA	1	Bulteel et al. 2020
		Social consequences of OSA (falling asleep, limited concentration…)	1	Broström et al. 2010
	Barriers	Lack of knowledge about OSA management and CPAP	10	Bakker et al. 2014; Berg et al. 2023; Broström et al. 2021(physician's point of view); Bulteel et al. 2020; Fung et al. 2017; Goldstein et al. 2022; Goyal et al. 2017; Khan et al. 2019; Nagarajan et al. 2012; Rezaie et al. 2018
		Suffering from insomnia	3	Goldstein et al. 2022; Wallace et al. 2013; Wickwire et al. 2010
		Laziness	2	Goyal et al. 2017; Nagarajan et al. 2012
		No OSA awareness/perception of symptoms	2	Goyal et al. 2017; Graco et al. 2019
		Perceived cognitive overload during the first visit	1	Bakker et al. 2014
		PTSD	1	Collen et al. 2012
		Type D Personality	1	Broström et al. 2007
		Lack of patient-centered approach	1	Broström et al. 2021 (physician's point of view)
		Depression	1	Guralnick et al. 2012
		Having a negative model	1	Rapelli et al. 2022
		Concern about image change	1	Ye et al. 2017
Precipitating	Barriers	CPAP-induced anxiety (claustrophobia, suffocation feeling)	10	Berg et al. 2023; Broström et al. 2010; Edmonds et al. 2015; Fung et al. 2017; Goldstein et al. 2022; Goyal et al. 2017; Khan et al. 2019; Khot et al. 2022; Wang et al. 2012; Ye et al. 2017
		Social embarrassment related to device use	6	Bakker et al. 2014; Broström et al. 2010; Goyal et al. 2017; Luyster et al. 2016; Møkleby et al. 2019; Rapelli et al. 2022
		CPAP-induced bed partners' anxiety	1	Bulteel et al. 2020
Perpetuating	Motivators	Perception of CPAP efficacy in symptoms resolution	13	Bakker et al. 2014; Berg et al. 2023; Broström et al. 2010; Bulteel et al. 2020; Dzierzewski et al. 2016; Fung et al. 2017; Graco et al. 2019; Khan et al. 2019; Khot et al. 2022; Sawyer et al. 2010; Møkleby et al. 2019 Luyster et al. 2016; Ye et al. 2017
		Feeling supported from technical provider/medical staff	6	Bakker et al. 2011; Bakker et al. 2014; Berg et al. 2023; Fung et al. 2017; Milinovic et al. 2024; Rapelli et al. 2022
		Feeling supported from bed partner	5	Berg et al. 2023; Gentina et al. 2019; Laharnar et al. 2024; Luyster et al. 2016; Ye et al. 2017
		Bed partner's CPAP efficacy perception	2	Bakker et al. 2014; Laharnar et al. 2024
		Awareness of the health benefits of CPAP	3	Bakker et al. 2014; Broström et al. 2010; Buckingham et al. 2020
		Self-efficacy	4	Dzierzewski et al. 2016; Sawyer et al. 2010; Wallace et al. 2013; Ye et al. 2012
		CPAP couple's benefits	2	Luyster et al. 2016; Sawyer et al. 2010
		Being supportive with the patients	1	Broström et al. 2021 (physician's point of view)
		Seeing oneself as a role model	1	Bakker et al. 2014
		High quality of marriage	1	Gentina et al., 2019
		No impediment in intimacy	1	Laharnar et al. 2024
	Barriers	Lack of support from technical provider/medical staff	5	Berg et al. 2023; Broström et al. 2010; Bulteel et al. 2020; Fung et al. 2017; Khan et al. 2019
		Impediment to intimacy	5	Berg et al. 2023; Luyster et al. 2016; Rapelli et al. 2022; Ye et al. 2012; Ye et al. 2017
		Suffering from insomnia	3	Goldstein et al. 2022; Wallace et al. 2013; Wickwire et al. 2010
		Perception of limited freedom	2	Broström et al. 2010; Møkleby et al. 2019
		Lack of support from the family	2	Goyal et al. 2017; Khot et al. 2022
		PTSD	1	Collen et al. 2012
		Type D personality	1	Broström et al. 2007
		Concern about image change	1	Ye et al. 2017

### Predisposing psychological factors in CPAP adherence

Predisposing psychological factors include both individual vulnerabilities that hinder CPAP adherence and personal strengths or contextual resources that promote it from the very beginning of therapy. These factors play a central role in shaping patients’ initial attitudes toward CPAP therapy and their likelihood of adherence. Among the most significant barriers is the lack of illness education and awareness, which encompasses limited understanding of OSA, its risks, and the benefits of CPAP therapy (Bakker et al., 2014; Berg et al., 2023; Broström et al., 2021; Bulteel et al., 2020; C. H. Fung et al., 2017a; L. A. Goldstein et al., 2022; Goyal et al., 2017; Khan et al., 2019; Nagarajan et al., 2012; Rezaie et al., 2018), coupled with a poor perception of symptoms, often leads to disengagement and low adherence (Goyal et al., 2017; Graco et al., 2019). To address this, patient education must prioritize clarity and accessibility, avoiding technical jargon in favor of simple, relatable language (Broström et al., 2021). Education should not merely aim to transfer information but help patients understand the connection between OSA, its potential health consequences (e.g., cardiovascular risks), and the protective role of CPAP (Broström et al., 2010). In this context, the design of psychoeducational interventions, including remote formats (Chen et al., 2020), aimed at enhancing patients’ knowledge of their condition, has been shown to promote better outcomes and improve self-efficacy (Chen et al., 2020; Tolson et al., 2023).

Another significant barrier to CPAP adherence involves individual psychological factors, such as Type D personality traits, characterized by high negative affectivity and social inhibition (Broström et al., 2007), Post-Traumatic Stress Disorder (PTSD) (Collen et al., 2012), depression (Guralnick et al., 2012), and concerns about changes in physical appearance (Ye et al., 2017). Depression, in particular, poses a critical challenge, as it can reduce motivation and discourage patients, especially when they do not experience immediate symptom relief (Weaver, 2019; Tolson et al., 2023). Screening for depression during the CPAP adaptation phase is essential, as undiagnosed or untreated depressive symptoms can exacerbate non-adherence, creating a cycle of frustration and perceived ineffectiveness of the therapy (Kerminen et al., 2019).

Proactive mental health screening by healthcare providers during this phase offers an opportunity to identify patients at risk and provide timely support. Integrating psychological interventions, such as counseling or support groups, into the treatment plan can foster a more supportive care environment (Poletti et al., 2024). This approach not only addresses underlying mental health challenges but also helps patients feel understood and engaged in their care journey, potentially enhancing their confidence and commitment to CPAP therapy.

Social factors also contribute to adherence challenges. Exposure to negative role models (Rapelli et al., 2022) and feelings of guilt or shame about the impact of OSA on the bed partner’s well-being can hinder engagement (Broström et al., 2010; Bulteel et al., 2020; Rapelli et al., 2022). However, involving the bed partner early in the treatment process can have a transformative effect. Bed partners, who often share the burden of disrupted sleep, can provide emotional and logistical support, creating a collaborative environment that fosters adherence (Gentina et al., 2019; Rosa et al., 2022). Open communication between patients and their partners further reduces feelings of guilt and shame, strengthening the support system (Møkleby & Mengshoel, 2019).

On the positive side, motivators such as health literacy (J. P. Bakker et al., 2011; Broström et al., 2010; Bulteel et al., 2020; C. H. Fung et al., 2017a; Fung, Martin, et al., 2017; Graco et al., 2019; Khan et al., 2019; Milinovic et al., 2024; Rapelli et al., 2022; Sawyer et al., 2010) and support from family and friends (J. P. Bakker et al., 2011; Broström et al., 2021; Gentina et al., 2019) significantly enhance adherence since the first stages of OSA management. When patients have a strong understanding of their condition, they are more likely to engage with treatment (Bulteel et al., 2020). A patient-centered approach by healthcare providers is equally crucial, fostering trust, self-efficacy, and confidence in managing the condition (Rapelli et al., 2021). Tailoring communication to individual preferences and actively involving patients in their care enhances adherence behaviors, as a foundation of trust often translates into sustained engagement with therapy (Buckingham & Corkeron, 2020).

**Fig. 2:**
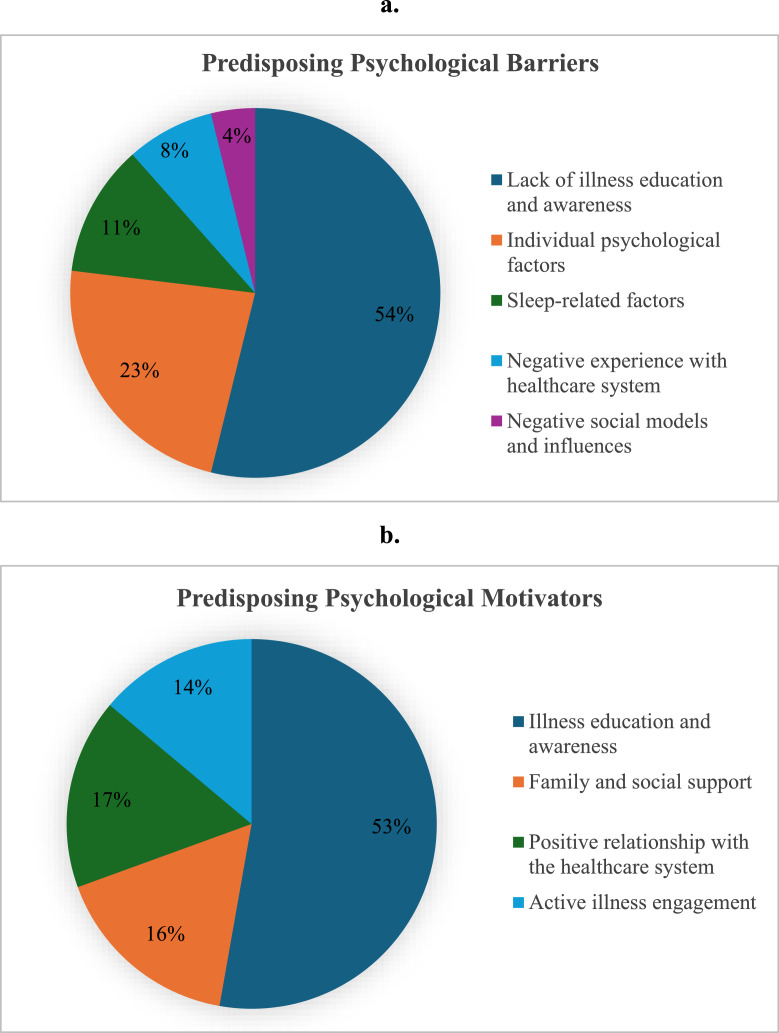
Predisposing psychological factors related to CPAP adherence (a. Barriers; b. Motivators).

Fig. 2 shows the frequencies of predisposing psychological barriers and motivators related to CPAP adherence.

### Precipitating psychological factors in CPAP adherence

Although precipitating factors can theoretically function as both facilitators and barriers, in the present review they emerged almost exclusively as acute psychological and social challenges occurring in the early phase of CPAP therapy, affecting both the patient and their bed partner. One of the most prominent barriers is CPAP-induced anxiety (J. C. Edmonds et al., 2015; Khot et al., 2022), often characterized by feelings of claustrophobia and fear of suffocation when using the mask. This anxiety can trigger a distress cycle, where the physical sensations of the mask provoke rapid breathing, chest tightness, and a heightened perception of suffocation (Wickwire et al., 2010). Over time, such experiences may lead to a conditioned negative association between the mask and feelings of panic, making subsequent use increasingly difficult (Edmonds et al., 2015).

Another significant challenge is social embarrassment tied to CPAP use (Møkleby & Mengshoel, 2019; Goyal et al., 2017), particularly in the presence of a bed partner. Patients often feel self-conscious wearing the mask, which can strain the shared sleeping environment. Visible struggles, such as difficulty adjusting to therapy or disrupted sleep, may evoke feelings of helplessness, worry, or even shame and guilt in the bed partner (Karademas, 2014). The physical presence of the CPAP machine, combined with its operational noise, can further amplify tension and discomfort within the couple, creating additional barriers to adherence (Bulteel et al., 2020).

To address these precipitating challenges, a dual-focused approach targeting both the patient and the bed partner is essential (Poletti et al., 2024). Open communication is a foundational strategy, helping bed partners understand the purpose and benefits of CPAP therapy while normalizing the potential difficulties during the adjustment period. Education for both parties about the therapy can alleviate fears, encourage shared responsibility, and foster a supportive environment that promotes adherence (Luyster et al., 2019).

Cognitive Behavioral Therapy (CBT) is a proven method to address CPAP-related anxiety (Sweetman et al., 2021). CBT equips patients and bed partners with tools to identify and challenge negative thoughts, such as perceiving the mask as "making things worse" (Richards et al., 2007). These thoughts can be reframed to focus on the long-term health benefits of therapy. Additionally, gradual desensitization techniques can help both individuals acclimate to the device and its presence in their shared environment, reducing anxiety over time (Friedberg et al., 2024; Espiritu et al., 2020).

Telemedicine adds another layer of practical support, allowing healthcare providers to address patient and bed partner concerns promptly. Virtual consultations can offer guidance on managing acute anxieties, resolving technical issues, and fostering open discussions to strengthen the partnership. This immediacy ensures that both parties feel supported, even outside traditional clinical settings (Labarca et al., 2021).

**Fig. 3:**
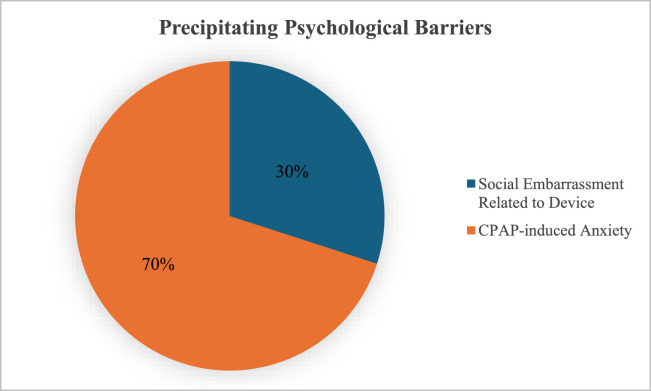
Precipitating psychological barriers related to CPAP adherence.

Incorporating a psychologist into the care process provides significant benefits (Tu et al., 2022). Psychologists can facilitate discussions between the patient and their bed partner, guiding them through the emotional and social impacts of CPAP therapy. By addressing these challenges collaboratively, psychologists can help reduce anxiety, enhance communication, and foster effective coping strategies. Such interventions not only improve adherence but also strengthen the couple’s resilience and emotional bond, creating a more supportive environment for long-term therapy success. Fig. 3 shows the frequencies of precipitating psychological barriers and motivators related to CPAP adherence.

### Perpetuating psychological factors in CPAP adherence

Perpetuating factors play a key role in the long-term course of CPAP adherence, with some elements contributing to disengagement and others supporting sustained use of therapy over time. Among these, insufficient healthcare support emerges as a major barrier. When patients encounter issues like mask discomfort, equipment malfunctions, or inadequate follow-up, they may feel unsupported and become discouraged, potentially discontinuing therapy (Berg et al., 2023; Broström et al., 2010; Bulteel et al., 2020; Khan et al., 2019). Regular and proactive engagement with healthcare providers is therefore essential to address these issues promptly, reinforce the benefits of therapy, and sustain motivation (J. P. Bakker et al., 2011; Berg et al., 2023; Milinovic et al., 2024; C. H. Fung et al., 2017a). For example, periodic follow-ups can provide opportunities for mask adjustments or troubleshooting, ensuring that patients remain comfortable and confident in using their device.

A lack of education about proper CPAP use and management further complicates adherence (Goyal et al., 2017), particularly when compounded by persistent sleep disturbances such as insomnia (Goldstein et al., 2022; Wallace et al., 2013; Wickwire et al., 2010). Tailored interventions, such as guided relaxation techniques (Wang et al., 2012), cognitive-behavioral strategies (Lichstein et al., 2013), or personalized mask refitting, can help patients overcome these barriers. Telemedicine offers a practical solution in this context, enabling remote resolution of technical issues while empowering patients to independently address minor challenges with the guidance of a technician (Labarca et al., 2021). This not only accelerates assistance but also fosters a sense of autonomy in managing therapy.

Psychological challenges, including depression, PTSD (Collen et al., 2012), or concerns about body image, present additional obstacles to CPAP adaptation. Relational dynamics further influence adherence, as the presence of a CPAP machine in the bedroom can evoke feelings of embarrassment or self-consciousness, potentially straining intimacy with bed partners (Berg et al., 2023; Luyster et al., 2016; Rapelli et al., 2022; Ye et al., 2012; Ye et al., 2017). Addressing these concerns through open communication and education is crucial. Involving bed partners in the treatment process—by equipping them with knowledge about OSA and the benefits of CPAP—can foster understanding and emotional support, which are vital for sustaining motivation (Guralnick et al., 2012; Kerminen et al., 2019; Laharnar et al., 2024).

On the positive side, several factors motivate patients to persist with CPAP therapy. Observable health improvements, such as reduced daytime fatigue, better sleep quality, and enhanced cardiovascular health, serve as powerful reinforcements, instilling confidence in the treatment’s effectiveness (Milinovic et al., 2024; Buckingham & Corkeron, 2020). Encouraging patients to monitor these improvements, perhaps through tools like symptom diaries (Tu et al., 2022) or health tracking apps (Baptista et al., 2022), can further bolster their sense of progress and self-efficacy.

The role of bed partners cannot be overstated (Luyster et al., 2016). Their active participation—providing reassurance during moments of frustration, assisting with practical aspects of therapy, and reinforcing the benefits of adherence—can be transformative (Barello et al., 2012). A collaborative approach to treatment reduces feelings of isolation and strengthens the patient’s resolve. For instance, healthcare providers might consider offering joint consultations or tailored psychoeducational sessions to involve both the patient and their partner (Karademas, 2014).

Finally, fostering a sense of agency in patients—helping them view themselves as active participants in their care—can have a profound impact. Patients who perceive CPAP adherence not only as a step toward personal health but also as an opportunity to model positive behaviors for their families or communities are more likely to remain committed to therapy (Bakker et al., 2011). This mindset shift, supported by consistent healthcare engagement and relational backing, lays the groundwork for sustainable adherence and improved quality of life.

**Fig. 4:**
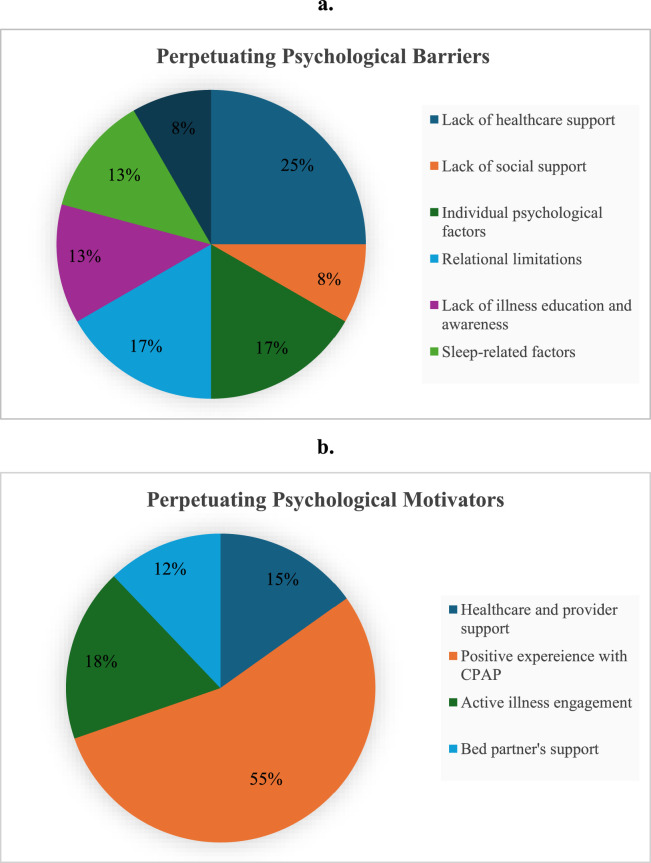
Perpetuating psychological factors related to CPAP adherence (a. Barriers; b. Motivators).

Fig. 4 shows the frequencies of perpetuating psychological barriers and motivators related to CPAP adherence.

### Non-psychological factors

As widely discussed in both literature and clinical practice, the analysis of the selected articles also revealed several non-psychological factors influencing CPAP adherence. Like their psychological counterparts, these factors were interpreted within the framework of the 3P Model, highlighting their role in treatment success.

Non-psychological barriers to CPAP adherence are often linked to practical and physical aspects of the treatment journey (Palm et al., 2021; El-Solh et al., 2010; Gagnadoux et al., 2011; Dzierzewski et al., 2016). Socioeconomic disadvantage is a major challenge (J. P. Bakker et al., 2011; Simon-Tuval et al., 2009), as limited access to necessary resources (Rezaie et al., 2018), including the cost of the device and follow-up visits, can hinder patient commitment to therapy. This underscores the importance of financial support programs (Tarasiuk et al., 2012) and healthcare policies that ensure access to essential treatments (Billings et al., 2011; (Choi et al., 2021).

Mask-related discomfort (J. P. Bakker et al., 2011; Khan et al., 2019; Nagarajan et al., 2012), such as skin irritation, dry mouth, or abdominal bloating, is another significant barrier, especially during the initial stages of adaptation. However, advancements in technology have led to the development of a wide range of masks designed to provide greater comfort and accommodate different facial shapes and lifestyles (Santos De Andrade et al., 2014). This makes the adaptation phase with the sleep technician a crucial stage: a thorough evaluation of the patient's needs and a well-informed choice of the most suitable mask model can prevent many difficulties, significantly improving tolerance and adherence.

Another important challenge is the distance from healthcare centers (Corrigan et al., 2022), which can limit access to regular technical and clinical support (Bakker et al., 2014). In this context, telemedicine offers a promising solution, allowing patients to receive timely assistance even remotely, particularly during critical moments or when technical issues arise. This approach not only enhances the sense of closeness and support but also reduces dropout rates by facilitating continuous monitoring of treatment (Labarca et al., 2021).

Among the motivators, full insurance coverage emerges as a key factor in reducing financial concerns (Daabek et al., 2021), allowing patients to focus on adherence. Additionally, retirement status, which provides more time and flexibility, facilitates managing appointments and medical visits, contributing to better treatment acceptance (Han et al., 2022).

## Discussion

The complexity of CPAP adherence in patients with OSA, as analyzed through the lens of the 3P Model framework, reveals a multifaceted interplay of psychological, social, and healthcare-related factors influencing adherence.

Predisposing factors encompass psychological barriers, such as limited illness awareness (Berg et al., 2023) and individual psychological traits (Broström et al., 2007), which can hinder motivation to adhere. Conversely, motivators within this domain emphasize the pivotal roles of illness education, tailored interventions, and family support in fostering adherence (Milinovic et al., 2024). Precipitating barriers, including social embarrassment, stigma, and CPAP-related anxiety (Goyal et al., 2017), further highlight the importance of addressing emotional and social dimensions of adherence. Perpetuating factors reflect challenges stemming from healthcare system limitations and relational difficulties, such as poor communication or inadequate follow-up (Bulteel et al., 2020). However, these barriers can be mitigated by the positive influence of supportive healthcare providers and reinforcing experiences of successful CPAP use, which help sustain long-term adherence. These barriers underline the necessity of adopting patient-centered approaches that normalize CPAP use and provide psychological support. Adherence to CPAP therapy extends beyond physical and economic constraints—particularly pressing in contexts without universal healthcare systems—to encompass the subjective experiences and beliefs of patients (DiMatte et al., 2012). In such settings, where access to diagnostic services, follow-up care, or equipment subsidies may be limited or inconsistently covered, patients may face additional structural barriers that intensify the psychological burden of initiating and maintaining therapy (Simon-Tuval et al., 2009). As previously noted (Poletti et al., 2023), beliefs and perceptions play a critical role in treatment adherence, even in severe respiratory conditions. Moreover, the support of bed partners and the relationship with healthcare providers emerge as pivotal factors (Laharnar et al., 2024). Bed partners are consistently recognized as key contributors to successful adherence (Baron et al., 2011), while healthcare providers play an essential role in guiding patients, fostering engagement, and promoting active involvement in the management of their condition (Barello et al., 2012). These relational dimensions should be central to any strategy aimed at optimizing CPAP adaptation and adherence.

### Integrative insights from the 3P model

This scoping review offers a comprehensive mapping of the psychological and behavioral factors influencing CPAP adherence in OSA patients. In doing so, it responds to the initial research question by illustrating how the 3P Model framework can be effectively applied to organize and interpret the complex array of adherence-related factors.

The interconnectedness of the factors identified in the 3P model—Predisposing, Perpetuating, and Precipitating—demonstrates the complexity of CPAP adherence and challenges the traditional concept of "compliance". Unlike compliance, which implies passive patient obedience to medical instructions, adherence requires active, conscious participation from the patient, shaped by a range of psychological, social, and environmental factors (De las Cuevas, 2011).

One such factor is the patient’s mental health, which plays a central role across all three categories (Guralnick et al., 2012). For instance, mental health issues such as depression or anxiety can act as predisposing factors, influencing the patient’s initial attitude toward CPAP therapy. These conditions can reduce motivation and foster negative thoughts about therapy, complicating the onset of adherence. However, mental health can also be a perpetuating factor. If depression or anxiety are not adequately addressed, they can undermine the patient's ability to cope with the inevitable challenges of CPAP therapy, such as mask discomfort or early treatment frustrations. Furthermore, during the early phases of treatment, these psychological challenges can act as precipitating factors—manifesting as anxiety over the mask or fear of suffocation, which may trigger distress and exacerbate difficulties with initial adaptation to the therapy (Collen et al., 2012; Edmonds et al., 2015).

The 3P model also demonstrates the role of a supportive environment, whether family or medical. As demonstrated by Luyster and collaborators (Luyster et al., 2016), an engaged and informed bed partner or family member can serve as a critical source of emotional support, not only preventing a negative mental health spiral but actively assisting in overcoming both perpetuating and precipitating barriers (Khot et al., 2022). For example, the bed partner’s encouragement during moments of distress or discomfort helps reduce the negative psychological feedback loop that might otherwise compromise treatment adherence (Laharnar et al., 2024). This collaborative support also acts as a predisposing factor, enhancing the patient’s initial willingness to engage with the therapy. Furthermore, continuous support from healthcare providers—offering regular check-ins, adjustments to the therapy, and guidance during difficult phases—can address both perpetuating and precipitating factors, ensuring that the patient feels consistently supported throughout the treatment process.

A key example of this interconnection lies in the patient's awareness of their condition and the role of CPAP in managing OSA. The patient’s initial understanding of their health, including the risks of untreated sleep apnea and the potential benefits of CPAP, functions as a predisposing factor (C. H. Fung et al., 2017a). However, when this awareness is not reinforced and nurtured throughout the treatment process, it can evolve into a perpetuating factor. Without sufficient knowledge, patients may become disengaged, fail to recognize the long-term benefits, or misunderstand the nature of their treatment (Goyal et al., 2017). In this way, patient education must be an ongoing process, ensuring that the patient maintains a high level of understanding and motivation over time (Graco et al., 2019). Moreover, if the patient’s condition is not improving as quickly as expected, frustration may trigger precipitating factors, such as anxiety or doubt about the therapy’s effectiveness. In this case, maintaining open communication and education at every stage of treatment is crucial to break the cycle of uncertainty (Rapelli et al., 2021). Importantly, some studies also suggest that psychological interventions, such as progressive muscle relaxation techniques, may enhance CPAP adherence by reducing anxiety and promoting a sense of control during treatment initiation. Wang and collaborators demonstrated that a combined intervention involving patient education and progressive muscle relaxation significantly improved adherence rates, as well as reduced depressive symptoms and treatment-related distress (Wang et al., 2012). These findings underscore the potential of integrating simple, low-cost psychological strategies into standard care to mitigate precipitating and perpetuating barriers to adherence.

These examples highlight how the same factors can play multiple roles throughout the treatment journey. It is this intricate interplay of psychological, social, and educational elements that underscores the complexity of CPAP adherence (Vermeire et al., 2001), demonstrating the need for a more active, informed, and collaborative approach to patient care. Adherence to CPAP is not merely a matter of passive compliance, but an ongoing, dynamic process shaped by the patient’s mental state (Collen et al., 2012), their social environment, and their evolving understanding of their condition. This perspective shifts the focus from the patient as a passive recipient of care to a more engaged partner in managing their health.

### Strengths, limitations and future directions

This review highlights several key strengths, including its innovative application of a psychological framework, typically used in other health contexts, to examine adherence to OSA treatment. Another notable strength is its focus on the subjective aspects of disease management, which are often overlooked in favor of clinical outcomes. By emphasizing the psychological factors that influence CPAP adherence, this review offers a more comprehensive understanding of the patient experience, extending beyond conventional biomedical approaches.

However, several limitations should be considered. The heterogeneity of the included studies, arising from the use of diverse methodologies without a focus on specific statistical outcomes, may affect the clarity of the findings. It is important to note that this broad approach was deliberately chosen to provide a comprehensive overview of psychological factors rather than to conduct rigorous quantitative analysis. To enhance clarity and precision, future research could focus on more homogeneous samples and specific statistical outcomes. In addition, this review did not consider changes in clinical indicators such as the evolution of the AHI over time, particularly as measured by CPAP device data. Nonetheless, a few studies included in the review (Milinovic et al., 2024; Gagnadoux et al., 2011)—such as Milinovic and collaborators, which reported follow-up AHI values, and Gagnadoux and collaborators, which identified AHI improvement as a factor associated with better adherence—did address the role of clinical outcomes. These findings, although relevant, were presented using heterogeneous methods and timeframes, and thus were not generalizable enough to be incorporated into the broader synthesis. Nevertheless, incorporating such clinical outcome measures in future reviews may help integrate psychological insights with objective markers of therapeutic response, offering a more holistic understanding of CPAP treatment trajectories.

Furthermore, future studies should investigate interventions that specifically address precipitating factors, such as CPAP-related anxiety, and examine longitudinal predisposing and precipitating motivators to understand how adherence-related factors evolve over time. Such research would contribute to the development of more tailored interventions, better suited to meet the changing needs of patients throughout their treatment.

### Conclusion

In conclusion, the concept of treatment adherence in the context of OSA is inherently multifactorial and requires the active involvement of both patients and their caregivers. Effective therapeutic approaches must be based on a patient-centered model, embedded within a biopsychosocial framework. In this approach, the psychological factors that influence treatment adherence and disease management are given equal importance to more objective variables. Recognizing and addressing the psychological dimensions of OSA treatment ensures a holistic perspective, promotes better patient engagement, enhancing adherence, and ultimately leads to improved health outcomes.

## Research funding

This research did not receive any specific grant from funding agencies in the public, commercial, or not-for-profit sectors.

## Declaration of competing interest

Eleonora Volpato declares the winning of an ERS Long-Term Research Fellowship 2024 grant (ID Number: LTRF202404-01155), which is unrelated to this study. The other authors declare that they have no competing interests.

## Appendix Supplementary materials

Image, application 1
